# Takotsubo syndrome in the setting of cardiac pacing: A review of mechanistic and clinical perspectives

**DOI:** 10.1007/s10741-026-10611-9

**Published:** 2026-03-20

**Authors:** Kenan YALTA, Ugur OZKAN, Ertan YETKIN

**Affiliations:** 1 Cardiology Department, Trakya University, Edirne, Turkey; 2 Cardiology Department, Şişli Kolan Hospital, Istanbul, Turkey; 3 School of Medicine, Cardiology Department, Trakya University, Balkan Yerleşkesi, Edirne, 22030 Turkey

**Keywords:** Takotsubo syndrome, cardiac pacing, clinical implications, mechanistic implications, permanent pacemaker implantation, conduction system pacing, intraventricular mechanical factors

## Abstract

Takotsubo syndrome (TTS) is a reversible form of acute cardiac syndrome usually arising in response to various emotional and physical stressors. TTS due to cardiac pacing has been an uncommon phenomenon mostly attributable to a variety of periprocedural conventional stressors including anxiety, pain and low cardiac output. Interestingly, intraventricular mechanical factors might also play a pivotal role in TTS evolution due to cardiac pacing. Notably, this phenomenon may be potentially underdiagnosed due to a variety of factors including atypical symptoms and masked electrocardiographic (ECG) changes potentially suggesting its higher incidence in clinical practice.

The present paper aims to focus on mechanistic and clinical implications of TTS due to cardiac pacing procedures including conventional permanent pacemaker implantation (PPI) and conduction system pacing (CSP) along with a particular emphasis on intraventricular mechanical factors as potential TTS triggers in this context.

## Introduction

 Takotsubo syndrome (TTS) is a reversible form of acute cardiac syndrome with a particular predilection for postmenopausal women [1–5]. Currently, adrenergic activation in response to various emotional and physical stressors has been regarded as central to the pathogenesis of this phenomenon [1–8]. This suggests a form of catecholamine toxicity at the myocardial level leading to a variety of adrenergic receptor and post-receptor alterations associated with oxidative stress, myocytolysis and myocardial stunning [1–8]. Furthermore, coronary spasm, coronary microvascular dysfunction as well as myocardial inflammation might potentially serve as adjuntive factors in TTS pathogenesis at least in a portion of cases [1–8]. TTS presents with various morphological patterns of myocardial involvement including apical (the most common variant), midventricular, basal, focal, global and even biventricular patterns [1–8]. Clinically, this phenomenon strongly mimics acute coronary syndromes (ACSs) in terms of its symptoms (including angina, dyspnea) and clinical findings (including electrocardiographic (ECG) findings such as ST segment elevation and T wave inversion along with elevated myocardial enzymes) [1–8]. Particular risk factors associated with unfavourable outcomes have been previously described in detail, and may be found elsewhere [3, 5, 6].

To date, various emotional or physical stressors have been reported as TTS triggers though spontaneous TTS episodes may also be likely in the clinical settting [1–5]. In this context, conventional permanent pacemaker implantation (PPI) was also reported as a potential TTS trigger in previous reports [9–30]. A variety of periprocedural stressors [9–30] as well as hemodynamic alterations associated with cardiac pacing [31, 32] might potentially induce TTS episodes in this context. Conduction system pacing (CSP) is a relatively novel and effective modality for cardiac pacing [33–35], and might also be associated with TTS evolution [36–39]. In this paper, we would like to highlight mechanistic and clinical implications of TTS in the context of cardiac pacing.

## TTS associated with conventional cardiac pacing

### Epidemiology

Conventional PPI was previously reported as a potential TTS trigger [9–30]. In general, TTS associated with conventional PPI has been an uncommon phenomenon [9]. In the RETAKO cohort, the percentage of PPI-related TTS was only 1.3% [9]. However, this phenomenon might be potentially underdiagnosed in clinical practice due to certain factors including atypical symptomatology and masked ECG changes [9, 10].

### Demographic and clinical features

In a recent multicentre prospective registry, patients with TTS due to conventional PPI (a total of 41 patients retrieved from the RETAKO registry and literature) were compared with patients with TTS due to other triggers (a total of 1559 TTS patients retrieved from the RETAKO registry) [9]. Indication for PPI was atrioventricular block (AVB) in the majority of patients with TTS due to PPI. Dual chamber pacemaker (right atrium (RA) and right ventricle (RV)) was implanted in most of these patients. The two groups differed in terms of certain clinical findings. Accordingly, the incidences of syncope (29,3% vs. 7.8%) and dyspnea (55.9% vs. 41.4%) were higher whereas the incidence of angina (29.3% vs. 64.8%) was significantly lower in patients with PPI-related TTS. More importantly, these patients were reported to have significantly lower left ventricular ejection fraction (LVEF) values (34.8% vs. 47.3%) and significantly higher rates of cardiogenic shock (20.6% vs. 8.8%), acute renal dysfunction (29.3% vs. 11.0%) along with longer corrected QT intervals (551.2 ms vs. 502.5 ms). However, the above-mentioned differences between the two groups were lost following propensity score matching and adjustment for potential confounders including physical trigger (and mix trigger) and baseline features. Of note, it was not feasible to detect ST-T wave changes in certain TTS cases due to paced beats [9].

However, there were no significant differences between the two groups (those with TTS due to conventional PPI vs. other triggers) in terms of all-cause mortality (12.2% vs. 13.1%), in-hospital mortality (2.4% vs. 2.0%), TTS recurrences (0.0% vs. 3.9%), age (predominantly elderly: 73.63 years vs. 70.52 years), sex (predominantly female: 78% vs. 86.7%) and morphological TTS patterns (predominantly apical involvement: 82.9% vs. 74.5%). Furthermore, the above-mentioned features did not significantly differ following propensity score matching [9].

It should be noted that the above-mentioned systematic review [9] is the largest analysis to date, and also includes the majority of patients in an earlier systematic review [10] analysing a total population of 28 TTS patients. Accordingly, these two systematic reviews [9, 10] share the same 18 cases in the literature. This earlier systematic review [10] also yielded similar demographic and morphological features along with similar TTS symptoms and pacing mode in patients with TTS due to conventional PPI (mostly female, mostly elderly, mostly atypical symptoms (or asymptomatic), mostly apical involvement and mostly dual chamber pacing). However, this earlier analysis [10] particularly highlighted the presence of prolonged TTS recovery, and reported a case fatality rate of 3.6%. Importantly, TTS onset was within the first 24 h following PPI in the majority of cases in both analyses suggesting a strong causal relation between PPI and TTS evolution in these cases [9, 10].

Of note, there are also a few reports not included in the above-mentioned systematic reviews. Among these, the first case of TTS due to a leadless pacemaker implantation was recently reported in an 84 year-old female patient [40]. A case of TTS due to an additional RV lead implantation (due to a lead fracture) was also reported in an 88 year-old female patient with a history of complete AV block requiring PPI [41]. These two patients [40, 41] also share the general features of TTS cases due to conventional PPI (elderly, female, with an apical balloning pattern exhibiting full recovery and in the context of high-degree AV block). TTS with an apical ballooning pattern following implantable cardioverter-defibrillator therapy (ICD) was previously reported in a 49 year-old male patient [42]. Finally, TTS with an apical ballooning pattern was reported in an 81 year-old female patient following conventional cardiac resynchronization therapy (CRT) [43].

### Clinical implications

**Fig. 1:**
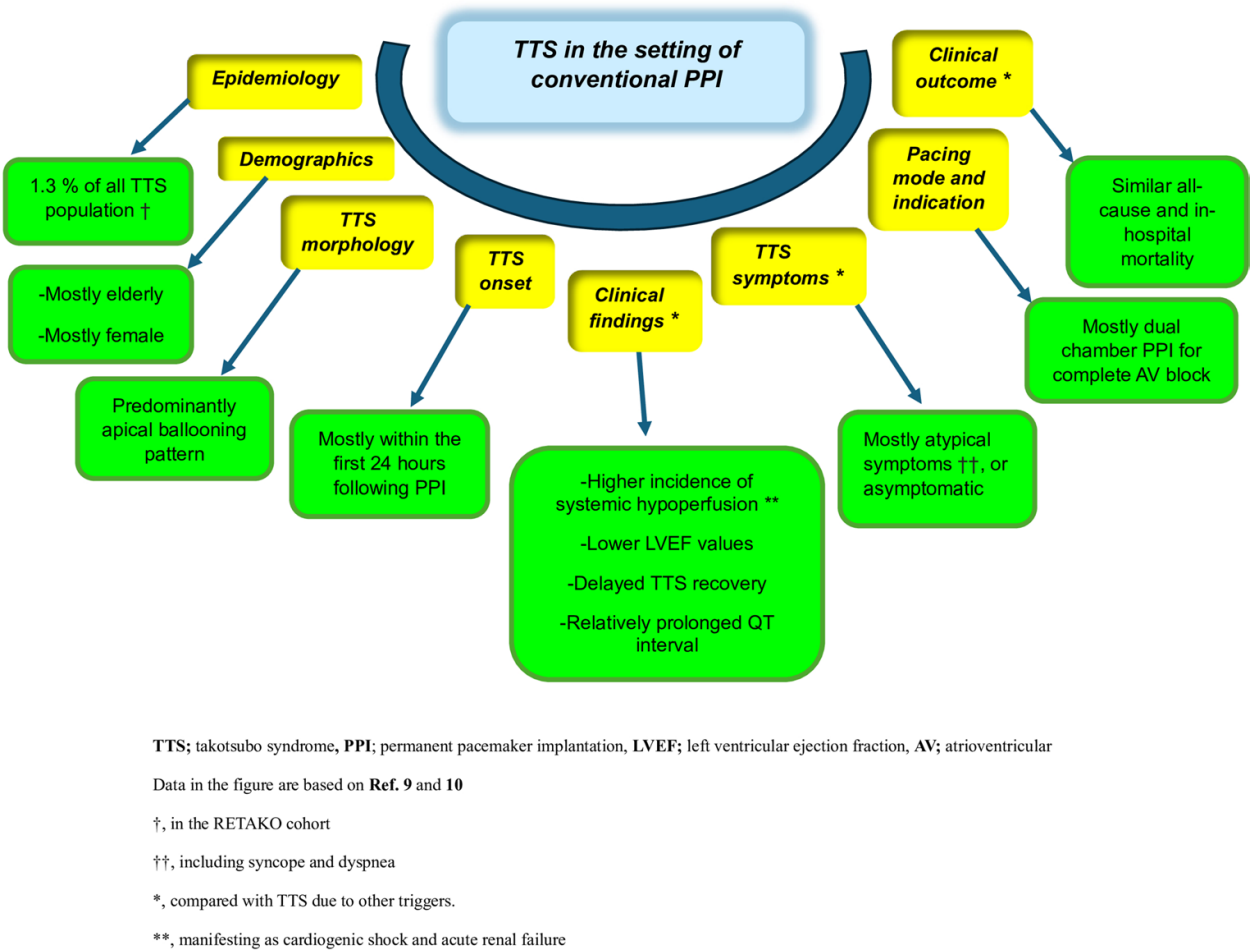
Summary of general features of takotsubo syndrome (TTS) due to conventional permanent pacemaker implantation (PPI)

It may be suggested that TTS due to conventional PPI have similar morphological and demographic characteristics compared with TTS due to other triggers [9, 10]. However, the higher incidence of atypical symptoms (even absence of symptoms in some patients) seems as a diagnostic challenge [9, 10]. Therefore, echocardiographic evaluation within the first day following the procedure should be the routine strategy following PPI in an attempt to uncover patients with vague or null symptomatology [9, 10]. It might also be suggested that lower LVEF values and associated clinical findings (including cardiogenic shock) [9] might not be due to the more extensive nature of TTS involvement, but could be attributed to additional adverse impact of conventional PPI on left ventricular (LV) systolic functions in such cases. Of note, the presence of relatively prolonged QT interval was suggested as a form of cardiac memory following paced beats in these patients [9]. Moreover, clinical differences in TTS cases due to PPI (including higher incidences of acute renal dysfunction and cardiogenic shock) might not be specifically related to cardiac pacing, yet; might primarily be associated with the physical nature of PPI as a TTS trigger and baseline characteristics of these patients [9]. Paced beats may potentially conceal ST-T segment changes in certain TTS patients due to PPI [9, 10], and may delay the TTS diagnosis. Demographic and clinical features of TTS due to conventional PPI are summarized in (Figure-1).

Finally, TTS episodes following ICD [42] and conventional CRT [43] have been extremely rare compared with TTS cases due to conventional PPI. This might possibly be attributable to a substantially higher likelihood of TTS underdiagnosis due to pre-existing systolic dysfunction in the overwhelming majority of these cases potentially hampering proper evaluation of characteristic wall motion abnormalities (WMAs) associated with TTS [2]. Moreover, clinical findings including dyspnea and elevation of myocardial enzymes might be potentially attributed to the pre-existing heart failure (HF) rather than new-onset TTS in this context.

### Mechanistic implications

A variety of emotional and physical factors (including anxiety, periprocedural pain, low cardiac output) have been suggested as potential stressors for TTS occurence in the setting of conventional PPI [9]. However, low cardiac output leading to adrenergic activation is more likely to arise as an outcome of severe bradyarrhythmias that prompt the clinicians to perform urgent PPI [9], and, hence, might not be regarded as a direct consequence of PPI. Low cardiac output may also arise due to acute exacerbation of pre-existing HF before or after device therapy. Importantly, device-related complications after PPI may also lead to low cardiac output state due to impaired ventricular filling in rare instances [31]. One such complication is ‘pacemaker syndrome’ that potentially emerges in the setting of single chamber (ventricular) pacing leading to atrioventricular dyssynchrony, loss of atrial contribution along with impaired ventricular filling with consequent low cardiac output [31] that might be associated with TTS evolution. However, majority of patients with PPI-related TTS were reported to receive dual chamber devices [9, 10]. Certain abnormalities in pacing parameters including abnormally short AV delay, AV dyssynchrony (other than pacemaker syndrome) as well as high intrinsic atrial rate in the VDD mode (leading to excessive tachycardia) may also lead to impaired ventricular filling leading to low cardiac output [31, 32].

However, it should be borne in mind that there might be alternative and more subtle mechanisms of low cardiac output including pacemaker-induced worsening in LV systolic functions. Accordingly, a high RV pacing burden (percent of paced beats) may potentially worsen LV systolic function (as measured with LVEF value) and cardiac output even in the setting of dual chamber pacing as a consequence of iatrogenic left bundle branch block (LBBB) pattern leading to interventricular and intraventricular dyssynchronny [31]. In this context, an RV pacing burden of > 40% has been suggested to be associated with worsening LV functions [10]. Progressive worsening of LV systolic function warrants upgrading to CRT [31] that constitutes biventricular pacing or novel strategies including CSP [33]. Conventional CRT (or CSP) instead of conventional PPI is already indicated in patients with low LVEF at baseline and a presumably high pacing burden. It should be borne in mind that malignant tachyarrhythmias, antitachycardia pacing and cardioversion (defibrillation) shocks (appropriate or inappropriate) might also serve as emotional and/or physical stressors in the setting of ICD or conventional CRT.

Finally, atropine and certain sympathomimetic agents including isoprenaline and dopamine may be of potential benefit in the context of symptomatic high-degree conduction blocks (including complete AV block) potentially reducing the need for temporary pacing strategies until PPI is implemented [44]. On the other hand, dobutamine has been regarded as an effective sympathomimetic agent used for the management of decompensated HF [44], and might occasionally be intiated in the periprocedural setting of ICD or CRT device implantation. However, administration of sympathomimetic agents is a well known TTS trigger as well [1], and hence might arise as an alternative mechanism of TTS evolution in the setting of cardiac pacing. In our clinical practice, we usually do not prefer to initiate sympathomimetic agents including isoprenaline (and prefer temporary pacing strategies instead) for the urgent management of high-degree conduction blocks due to the life-threatening adverse affects of these agents including ventricular arrhythmias, hypotension as well as induction of coronary ischemia and TTS [44]. In summary, it seems likely that a combination of stressors, rather than a single stressor, might account for the development of TTS in the setting of conventional cardiac device therapy.

## TTS and CSP

### CSP: Basic aspects

CSP is a relatively novel and technically demanding pacing strategy with important technical tips and tricks as well as clinical implications [33–35]. This strategy requires deep myocardial tissue penetration for proper lead implantation [33–35]. CSP mostly encompasses His bundle pacing (HBP), left bundle branch (LBB) pacing and left fascicular pacing (LFP) primarily aiming to create a more physiological pacing pattern [33–35]. Terminologically, LBB area pacing denotes septal myocardial or LBB pacing (or LFP). In the clinical setting, CSP might be utilized as a strategy for CRT in the setting of advanced HF or as an alternative mode of cardiac pacing for the management of bradyarrhythmias [33–38].

In patients with HF with a CRT indication, CSP was found to be associated with a more physiological ventricular activation pattern, more favorable hemodynamic and echocardiographic improvement along with better clinical outcomes compared with conventional CRT (biventricular) [34]. Moreover, CSP in the setting of HF may be implemented together with conventional CRT (hybrid strategy in selected cases) [34].

**Fig. 2:**
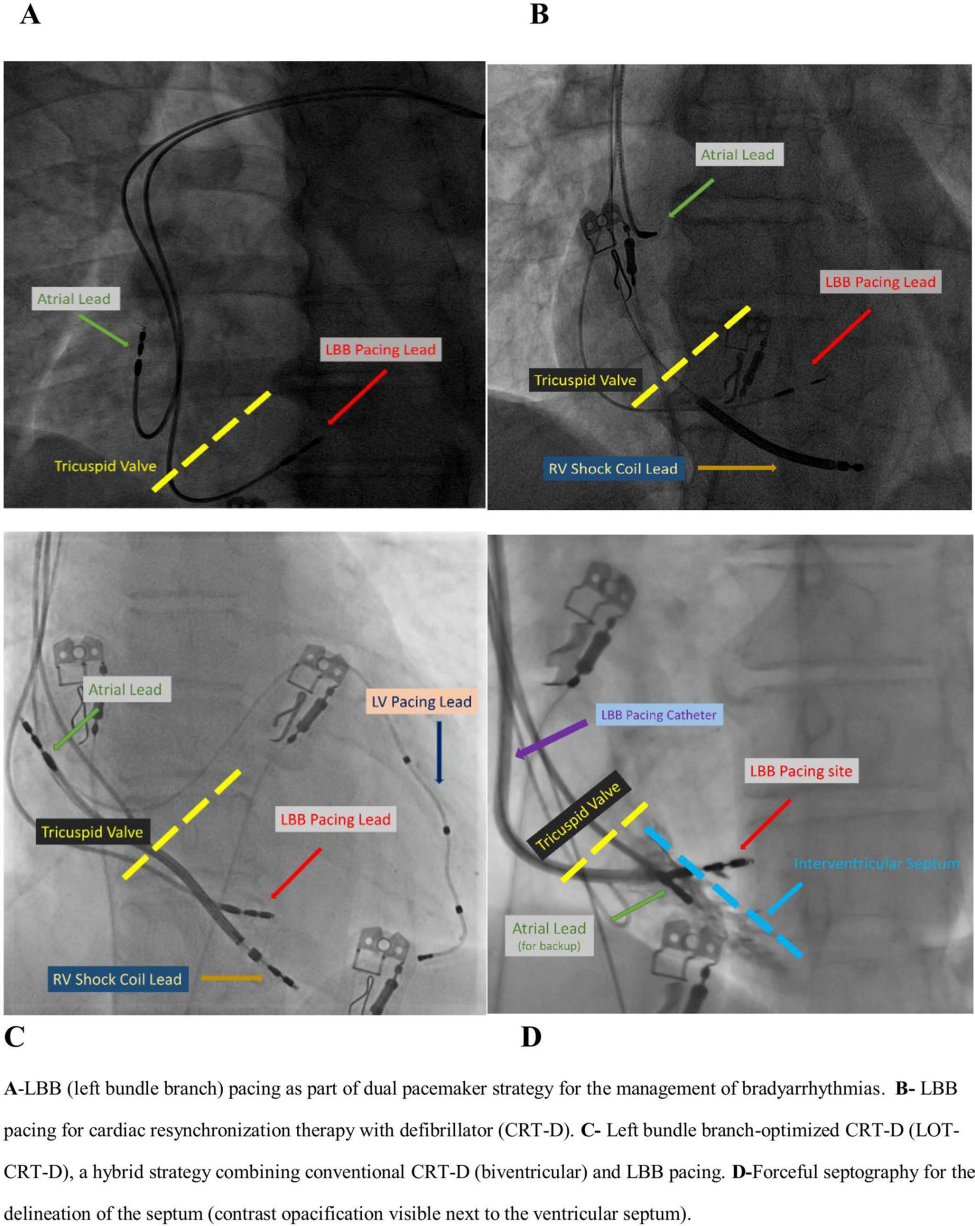
CSP for various clinical indications (**A**, **B**, **C**) and septography (**D**)

In any CSP strategy, deep septal myocardial penetration (necessary for proper lead implantation), excessive catheter manipulation as well as repetitive attempts for lead implantation [33–35] might be associated with important clinical consequences. Figure-2 (A, B, C) demonstrates the utility of CSP for various clinical indications. Detailed information on CSP may be found elsewhere [33–35], and is beyond the scope of this paper.

### Clinical features and implications of TTS in the setting of CSP

Notably, data regarding TTS occurence in the setting of CSP are scarce, and mostly based on case reports [36–39]. The first case of CSP-induced TTS was reported in a 93 year- old male patient who developed apical ballooning and left ventricular outflow tract obstruction (LVOTO) after LBB pacing [36]. Similar to the general TTS population, these cases were elderly and mostly female (3 patients) and have a morphological pattern of apical ballooning (in all cases) [36–39]. TTS was reported to occur following LBB area pacing in all cases [36–39]. Interestingly, symptoms other than angina including dizziness, dyspnea and pallor have been the predominant symptoms in these TTS cases [36–39]. Demographic and clinical characteristics of these published cases have been presented in (Table-1).

**Table 1:** Summary of published cases with TTS due to conduction system pacing (CSP)

	Age	Gender	Indication for CSP	CSP strategy	TTS symptoms and signs	ECG findings	TTS morphology and LV dysfunction	CAG findings	Clinical outcome
Scuotto F, et al. (36)	93 y	Male	Previous pacemaker malfunction	LBB pacing.Dual chamberwith the exclusive replacement of the previous RV lead.	Pallor, shock, signs of hpoperfusion following the anaesthesia withdrawal	T wave inversion in precordial leads	Apical ballooning pattern.LVEF: 15%LVOTO (pressure gradient: 83 mmHg), moderate-severe MR	Non-obstructive, previous stent implantation in the LAD artery	TTS recovery,Mortality due to pneumonia
Korpysz A, et al. (37)	74 y	Female	Complete AVB	LBB area pacing (left posterior fascicle capture).Dual chamber.	Dyspnea, hypotension, hypoxemia, tachycardia at 12 hours following discharge (discharged the following day after the procedure)	Atrial tachycardia, ST segment elevation in the leads V4-V6 followed by T wave inversion and QT prolongation.	Apical hypokinesisLVEF: 30%	Non-obstructive	Complete recovery of WMA and ECG at 10 weeks
Thaitirarot C, et al (38)	62 y	Female	2:1 AVB	LBB pacing.Dual chamber	Dyspnea, dizziness, chest tightness at 2 hours following the procedure	Mild ST elevation and T wave in version in the leads V2-V6 on day 3 following CSP.	Apical ballooning. LVEF: 35-40 %	Non-obstructive. Focal haziness in the proximal LAD on CAG. OCT confirmedan eccentric plaque of < 40% in the same area.	Discharged on day 5 with partial TTS recovery.
Li YP, et al (39)	89 y	Female	Complete AVB	LBB area pacing.Dual chamber.	Agitation and chest pain within 30 minutes following the procedure	Diffuse ST elevation, 2:1 AVB when pacing was set to 30 b.p.m.	Apical ballooning.LVEF: 45%	Non-obstructive	Discharged on day 9

*CSP* conduction system pacing, *TTS* takotsubo syndrome, *ECG* electrocardiogram, *LV* left ventricle, *CAG* coronary angiogram, *LBB* left bundle branch, *RV* right ventricle, *LVEF* left ventricle ejection fraction, *LVOTO* left ventricle outflow tract obstruction, *LAD* left anterior descending, *AVB* atrioventricular block, *OCT* optic coherence tomography

In summary, these cases are similar to those with TTS due to conventional PPI (and also the general TTS population) in terms of morphological and demographic features [36–39]. In-hospital outcome also seems favorable in this context, and the mortality reported in 1 patient was not directly attributable to TTS (pneumonia) [36]. Interestingly, all the cases reported were due to LBB area pacing [36–39]. This may suggest that LBB area pacing might have a higher risk of TTS development compared with His pacing. However, this might also be due to the significantly higher number of cases undergoing LBB area pacing in routine practice. Finally, all the cases had overt ECG findings during the onset and course of their TTS episodes [36–39]. This is in line with the notion that CSP enables proper evaluation of ST-T segment changes during paced beats [37] due to its physiological activation pattern [33–35].

### CSP as a TTS trigger: More pronounced impact of periprocedural conventional stressors

Since CSP restores and maintains synchronous ventricular activation pattern [33–35], CSP-related low cardiac output is unlikely unless a device or lead-related malfunction arises. However, pacing parameters (including AV delay) should be properly adjusted for optimal ventricular filling [32]. Notably, previously-mentioned emotional and physical stressors (including anxiety and periprocedural pain) in the context of conventional PPI might also arise in the setting of CSP, even to a greater extent, possibly due to the longer procedure and fluoroscopy times of this pacing strategy [45–48].

In a recent analysis comprising 442 patients, procedure and fluoroscopy times were found to be significantly higher in CSP cases compared with conventional PPI cases particularly in those receiving dual chamber implants, but not in those receiving single chamber or CRT devices [45]. On the other hand, operator experience might also significantly matter in terms of procedure and fluoroscopy times in the setting of CSP [46]. Accordingly, procedure and fluoroscopy times were reported to decrease steeply (with increasing success rates) reaching a plateau after performing 150 cases of LBB area pacing for the management of bradyarrhythmias [46]. During this stable stage of the operator (following 150 cases), a significant, yet; slight difference between CSP and conventional PPI with regard to procedure and fluorscopy times were still existent. However, this seems quite trivial compared with those during the initial experiences of the operators [46]. Similarly, earlier studies also demonstrated longer procedure and fluoroscopy times [47, 48] along with higher levels of post-procedural troponin T levels in the setting of LBB area pacing compared with conventional PPI [47].

In summary, the above-mentioned findings [45–48] suggest that CSP, despite its favorable impact on cardiac output and hemodynamics, is a complex procedure potentially acting as a significant physical stressor. Nevertheless, LBB area pacing has been associated with shorter procedure and fluoroscopy times along with better pacing parameters compared with HBP, and might be regarded as the preferred CSP strategy [46].

## Intraventricular mechanical factors: Alternative yet; underrecognized triggers of TTS evolution due to cardiac pacing

Based on previously published cases, there has been no single strong trigger that might absolutely account for the induction of a particular TTS episode in patients undergoing PPI [36] potentially suggesting the impact of subtle stressors in combination or alternative mechanisms of TTS evolution in these patients. Interestingly, intraventricular mechanical factors might arise as alternative mechanisms with important pathogenetic implications in the context of TTS associated with cardiac pacing.

### Mechanically-triggered TTS: Definition and general implications

TTS due to intraventicular mechanical factors was previously termed ‘mechanically triggered TTS’ [3]. Accordingly, TTS exclusively in the form of apical ballooning pattern may arise as a mechanically-triggered phenomenon due to sudden increases in intraventricular gradient leading to supply-demand mismatch and myocardial stunning in the LV apical territory in patients with hypertrophic cardiomyopathy (HCM), hypertensive myocardial disease or existing ventricular septal bulge [3, 49–51]. However, severe myocardial hypertrophy has a protective role against the evolution of apical ballooning in this context [3, 49–51]. Moreover, this form of apical ballooning pattern arises regardless of co-existing adrenergic discharge (despite its potential role in the evolution or aggravation of intraventricular gradient in some cases) [3]. Sudden intraventricular obstruction may be in the form of LVOTO or midventricular obstruction (MVO) [3]. In general, a peak gradient of > 50 mmHg (at rest or provoked in response to certain physiological maneuvers (including Valsalva maneuver) or hemodynamic stressors leading to reductions in preload and/or afterload and increased myocardial contractility through mechanisms including tachycardia) has been regarded as a clinically significant gradient in this context [3]. Intraventricular gradient, to a lesser degree, may be already evident at rest, and may be further provoked in the setting of HCM [3]. However, intraventricular gradient is usually absent at rest in the setting of ventricular septal bulge [52].

In particular, LVOTO may also arise as the consequence of a particular TTS episode (a potential complication of apical ballooning pattern) [3]. However, LVOTO as a TTS complication is more lenient, and completely resolves upon TTS recovery [3]. Therefore, particular causative role of LVOTO in TTS evolution should be established through clinical algorithms one of which has been recently demonstrated in the latest TTS consensus report [3].

### Hemodynamic stressors associated with cardiac pacing may induce mechanically-triggered TTS episodes in the presence of pre-existing myocardial disease

As expected, emergence of hemodynamic stressors may be quite likely in the post-cardiac pacing setting. For instance; certain abnormalities in pacing parameters (conventional PPI or CSP) including abnormally short AV delay, AV dyssynchrony or high atrial rate in the VDD mode may elicit reductions in LV filling and afterload along with tachycardia [31, 32], and may induce the development of mechanically-triggered TTS episodes in those with hypertensive myocardial disease, HCM or ventricular septal bulge [3]. Even an episode of severe vagotonia during the periprocedural stage may also result in significantly reduced afterload leading to intraventricular obstruction in the setting of cardiac pacing. However, conventional PPI in the form of apical RV pacing has been traditionally suggested to mitigate LVOTO in the setting of HCM largely through mechanisms including delayed and paradoxical septal activation pattern (and hence; recommended as a therapeutic modality in this context) [32]. Therefore, this paradoxical septal motion due to apical RV pacing may, to some extent, counterbalance the adverse effects of cardiac pacing on LVOT gradient.

It seems noteworthy that the above-mentioned myocardial abnormalities are quite prevalent in the general population [52]. HCM was found to be particularly more common in females after the age of 60 years [52]. Moreover, the prevalence of ventricular septal bulge (characterized by the exclusive presence of basal septal hypertrophy with a sigmoid septum pattern) increases with age potentially ranging from 4% to 8% in subjects of ≥ 60 years of age, and even reaching 10% in the octogenerians [52]. Notably, patients with these myocardial abnormalities and previously reported patients with TTS due to cardiac pacing have similar demographic features [9–30]. This may suggest a relatively higher incidence of pre-existing myocardial abnormalities in those with TTS due to cardiac pacing. However, pre-existing myocardial abnormalities and their association with mechanically-triggered TTS episodes were not specifically mentioned in the previous systematic reviews and case reports on TTS due to cardiac pacing [9, 10]. Finally, sudden intraventricular obstruction as a TTS trigger was previously suggested to be more prevalent than expected in the general TTS population with an apical ballooning pattern potentially labeling this phenomenon as an alternative theory in TTS pathogenesis regardless of pre-existing myocardial disease [53].

### Direct impact of CSP on the induction of mechanically-triggered TTS episodes: A speculation that needs to be established

It seems possible that the impact of physiological pacing strategies (including CSP) on the interventricular septum may directly induce mechanically-triggered TTS episodes regardless of emerging hemodynamic stressors and pre-existing myocardial disease. As a speculation, CSP may have the potential to transiently induce or aggravate LV intraventricular gradient possibly as a consequence of procedure-related septal trauma leading to potential alterations in the interventricular septal myocardium (including morphological changes and hypercontractility). Acute myocardial response to stretch characterized by hypercontractility is a well known phenomenon largely mediated through calcium (Ca) dependent mechanisms [54]. Moreover, myocardial manipulation during technically demanding cardiac interventions including cardiac pacing was previously suggested as a potential mechanical trigger of TTS episodes [41]. Accordingly, the first reported case of CSP-related TTS describes a 93 year-old man suffering TTS and LVOTO following LBB pacing [36]. This case might have been a mechanically-triggered TTS episode possibly attributable to a sudden LVOTO induced by the CSP-related septal trauma and/or pacing-related hemodynamic stressors (including tachycardia and reduction in preload) [36]. However, LVOTO in this case might have also risen as a consequence of TTS [36].

Mechanistically, CSP-related mechanical septal trauma may be ascribed to the screwing of CSP lead with an extreme torque build-up (potentially associated with endocardial entanglement), tunnel formation, forceful contrast injection (for septography) (figure-2-D) and repetitive maneuvers for proper lead implantation [33]. During the procedure, lead-related trauma may also be evidenced by ectopic ventricular beats (namely fixation beats) arising from the area of lead penetration [33]. Furthermore, mechanical septal trauma may also result in subtle complications with a mass effect including transient septal hematoma which might potentially impinge on the LVOT or RV outflow tract (RVOT) [33, 55–57]. In this context, septal hematoma was suggested to be associated with lead implantation in the high anteroseptal (that has a close proximity to LVOT territory) and low posteroseptal areas where large septal artery branches exist [55]. Conservative management [55, 56] and percutaneous coil embolization of the septal artery [57] have been recommended as the reasonable management strategies largely depending on the clinical features of the septal hematoma. Notably, the emergence of procedure-related sudden intraventricular obstruction may be significantly facilitated in the presence of pre-existing septal bulge or HCM with a subtle gradient.

**Fig. 3:**
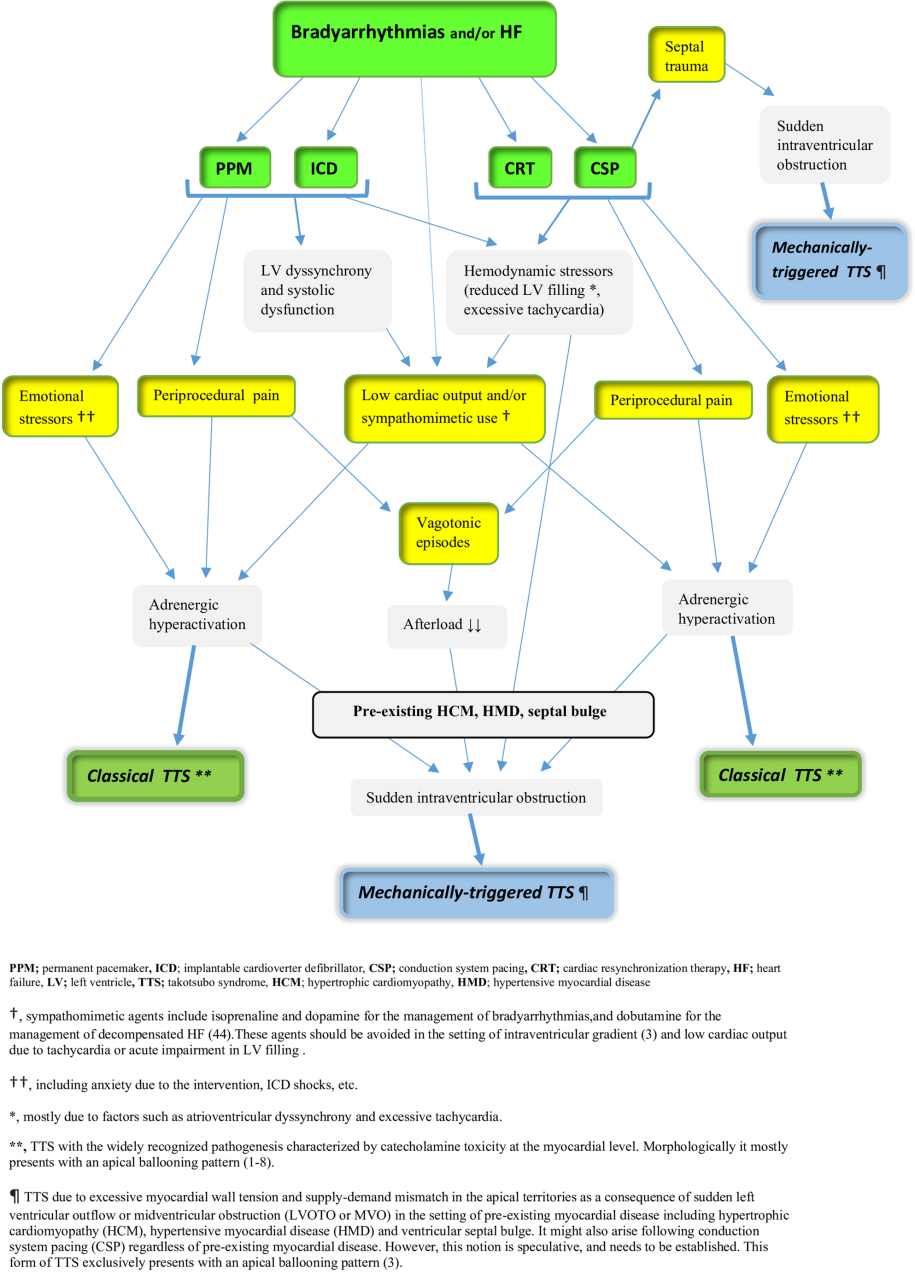
Potential stressors and mechanisms (and their complex interplay) in the context of TTS due to cardiac pacing

In summary, a variety of periprocedural stressors with certain mechanisms might play a pivotal role in the genesis of TTS episodes associated with cardiac pacing (Figure-3). Moreover, there might exist a complex interplay among these triggers and mechanisms.

Finally, it should be noted that emerging regional myocardial contractile dysfunction in the post-PPI setting may not always denote a true TTS episode potentially creating a diagnostic challenge. Accordingly, a high burden of RV apical pacing may occasionally lead to a pattern of overt apical contractile dysfunction as a consequence of early electrical activation of the apical myocardial regions (including LV apex) along with a compensatory hypercontraction pattern in the late activated areas remote from these regions [58, 59]. Morphologically, this pattern of pacing-induced apical contractile dysfunction may potentially mimic TTS leading to a TTS overdiagnosis in certain patients. In this context, ECG changes (including memory T wave inversion) and elevation of cardiac biomarkers, both of which are quite likely in the post-PPI setting, may also contribute to this overdiagnosis. Of note, this pathological pattern may be clinically silent in the initial post-PPI period and, unlike the apical ballooning pattern of a true TTS episode, generally appears to be persistent unless the pacing lead is repositioned in another myocardial site. Moreover, it may lead to global LV dysfunction in the long term due to progressive myocardial hypertrophy in the late activated LV regions as well as dyssynchronous LV contraction pattern [58, 59].

## Preventive measures and management strategies in the setting of TTS due to cardiac pacing

Conventional periprocedural stressors including anxiety and pain should be handled carefully in every patient undergoing PPI procedure. Accordingly, these stressors might be managed with anxiolytics, analgesics (local and systemic) and proper sedation. Proper intravenous hydration along with the avoidance of hypotension and tachycardia during the periprocedural period may mitigate the adverse impact of previously-mentioned hemodynamic stressors associated with the induction of mechanically-triggered TTS episodes [3]. Pacing mode and parameters (including AV delay) should be properly adjusted [32]. In particular, every effort should be made to minimize the mechanical trauma impinged on the ventricular septum during the CSP procedure which is characterized by a gradual learning curve in clinical practice [33–35]. Importantly, patients undergoing conventional PPI or CSP procedure should be evaluated on echocardiogram within the first day and in case of new-onset symptoms following the procedure [9, 10, 55–57]. Since a significant portion of patients with pacing-related TTS might be asymptomatic [10], serial re-evaluation on echocardiogram (on a daily basis) during the hospital course might be a prudent and reasonable strategy particularly following the CSP procedure. These measures enable timely diagnosis and management of a possible TTS episode, new-onset complications (including septal hematoma) as well as intraventricular obstruction.

However, it should be borne in mind that a significant portion of patients with a mechanically-triggered TTS episode have no overt intraventricular gradient on diagnostic cardiac imaging for TTS suggesting the transient and dynamic nature of this gradient [3]. In those with a significant gradient at rest, hydration, alpha agonists (including phenylephrine), up-titration of beta blockers have been recommended during the acute TTS course [3]. In resistant cases with pulmonary edema or cardiogenic shock due to LVOTO, mechanical support devices and rarely urgent septal ablation may be performed [3]. In patients with morphological features of HCM or hypertensive myocardial disease on echocardiogram, yet; without a significant intraventricular gradient at rest, certain provocation tests including exercise or dobutamine stress echocardiogram may be performed following complete TTS recovery in an attempt to uncover any significant intraventricular gradient [3]. As long-term management strategies, initiation of certain medications including beta blockers, non-dihydropyridine calcium channel blockers, disopyramide and novel myosin ATPase inhibitors (including mavacamten) as well as implementation of more radical options including percutaneous or surgical septal reduction therapies have been associated with symptomatic benefit in the context of HCM with a significant LVOTO (at rest or provoked) [3, 50, 60]. Accordingly, these management strategies also have the potential to prevent future mechanically-triggered TTS episodes [3] due to cardiac pacing or other stressors largely based on their favorable impact on the mitigation of intraventicular gradient [50, 60]. Potential preventive measures and management strategies in the context of TTS due to cardiac pacing have been summarized in (Table-2).

**Table 2:** Preventive measures and management strategies in the context of TTS due to cardiac pacing

Proper management of periprocedural pain and anxiety (analgesics, anxiolytics and sedation)
Periprocedural intravenous hydration †
Proper adjustment of pacing mode and parameters (including AV delay)
Avoidance of hypotension, dehydration and tachycardia
Avoidance of hypotension, dehydration and tachycardia
Avoidance of excessive septal trauma during the CSP procedure ††
Echocardiographic evaluation within the first 24 hours and in case of new-onset cardiovascular symptoms following cardiac pacing procedure *
Further evaluation with provocation tests (including dobutamine or exercise stress;echocardiogram) in the post-PPI setting in patients with confirmed or suspected HCM, HMD or ventricular septal bulge; yet without an overt intraventricular gradient at rest **
Mitigation of significant intraventricular gradient (at rest or provoked) for the prevention of future mechanically-triggered TTS episodes induced by cardiac pacing or other stressors ¶

*AV* atrioventricular, *CSP* conduction system pacing, *HCM* hypertrophic cardiomyopathy, *HMD* hypertensive myocardial disease.†,  This strategy may counterbalance possible hemodynamic stressors including reductions in preload and afterload along with tachycardia and associated low cardiac output. Preprocedural hydration also leads to venous distention, and facilitates subclavian vein puncture potentially preventing repetitive attempts. Therefore, this strategy may also reduce procedural time as well as anxiety and pain of the patient.  ††, Excessive trauma may result in sudden intraventricular obstruction through various mechanisms including enhanced myocardial contractility. However, this is speculative, and needs to be established.*, For the timely diagnosis of TTS, possible intraventricular gradient and other complications.**, This may confirm the presence of significant dynamic intraventricular gradient. In patients suffering a TTS episode in the post-PPI setting, these provocation tests should be performed following complete TTS recovery (3).¶, Intraventricular gradient in the long-term may be managed with certain medications including beta blockers, non-dihydropyridine calcium channel blockers, disopyramide and novel myosin ATPase inhibitors (including mavacamten)  as well as certain radical strategies including percutaneous or surgical septal reduction therapies (60). These management strategies potentially prevent acute rises in intraventricular gradient and hence; future mechanically-triggered TTS episodes due to cardiac pacing or other stressors (3).

## Conclusion

TTS due to cardiac pacing has been an uncommon phenomenon in the current literature possibly suggesting its underdiagnosis due to a variety of factors including higher incidence of atypical symptoms, masked ECG changes, and pre-existing HF. In general, patients with TTS due to cardiac pacing have similar demographic and morphological features compared with the general TTS population. Despite the higher proclivity for certain adverse outcomes, these patients have similar all-cause mortality. Temporally, TTS mostly arises within the first day following cardiac pacing procedures suggesting a strong causal relation between these procedures and TTS occurence in this context.

Mechanistically, periprocedural conventional stressors (including anxiety, pain, low cardiac output and sympathomimetic use) usually in combination have been suggested as potential TTS triggers in the context of cardiac pacing. However, intraventricular mechanical factors including sudden intraventricular obstruction (in response to hemodynamic stressors) may also arise as alternative, yet; underrecognized mechanisms of TTS evolution in the setting of cardiac pacing. This form of mechanically-triggered TTS episodes have been mostly encountered in those with pre-existing myocardial conditions including ventricular septal bulge, HCM and hypertensive myocardial disease all of which are quite prevalent in the elderly population. As a speculation, CSP may directly lead to sudden intraventicular obstruction largely due to its potential effects on the ventricular septum.

Finally, certain preventive measures (including periprocedural hydration, reduction of periprocedural pain and anxiety along with the avoidance of hemodynamic stressors) may significantly reduce the risk of TTS evolution due to cardiac pacing. Echocardiographic evaluation within the first day following pacemaker implantation should be the routine strategy for the timely diagnosis of TTS and pacing-related complications. Further aspects of TTS due to cardiac pacing still need to be established through large scale studies.

## Data Availability

No datasets were generated or analysed during the current study.
